# Integrative microbiome–methylome analysis links bacterial taxa to colorectal cancer histological subtypes

**DOI:** 10.1128/msystems.00622-26

**Published:** 2026-08-24

**Authors:** Ángel Escudero-Jiménez, Jorge Galán-Ros, Francisco Huertas-López, Andrea Pérez-Sarabia, Diogo Ribeiro, Fátima Postigo-Corrales, Ana Albaladejo-González, María Isabel Díaz-López, María Dolores López-Abellán, José García-Rodríguez, María Asunción Beltrán-Videla, Ana Belén Arroyo, Ana María Hurtado López, Ana Tapia-Abellán, Rubén Corral San Miguel, Edith Rodríguez-Braun, Eduardo Feliciangeli, Alberto Sánchez-Espinosa, Ana Conesa, Ginés Luengo-Gil, Pablo Conesa-Zamora

**Affiliations:** 1 Department of Microbiology and Parasitology, Complejo Hospitalario Universitario de Albacete 16242, Albacete, Spain; 2 Servicio de Microbiología y Parasitología, Hospital Clínico Universitario Virgen de la Arrixaca 16518 , Murcia, Spain; 3 Group of Molecular Pathology and Pharmacogenetics, Instituto Murciano de Investigación Biosanitaria, Hospital General Universitario Santa Lucía 525168, Cartagena, Spain; 4 Marbyt—Smart Solutions for Biotechnology S. L., Murcia, Spain; 5 Group of Genetics, Molecular Pathology and Rare Diseases. Universidad Católica de Murcia (UCAM), Guadalupe, Spain; 6 Group of Immunology, Department of Biochemistry and Molecular Biology (B) and Immunology, Campus de Ciencias de la Salud, Universidad de Murcia 16751, Murcia, Spain; 7 Genomics of Gene Expression Lab, Institute of Integrative Systems Biology, Spanish National Research Council (CSIC-UV) Catedràtic Agustín Escardino Benlloch, Paterna, Spain; APC Microbiome Ireland, Cork, Ireland

**Keywords:** microbiome, methylation, colorectal cancer

## Abstract

Colorectal cancer (CRC) arises through distinct molecular and histological routes that may be shaped by interactions between the mucosa-associated microbiota and host epigenetic regulation. We analyzed paired tumor and adjacent non-tumor colorectal mucosa from CRC patients stratified by histological subtype (conventional vs serrated-pathway groups). Microbiota composition was profiled by Illumina sequencing, and host DNA methylation was assessed using genome-wide CpG arrays with targeted validation. Alpha diversity showed modest tumor–non-tumor differences, with variation by anatomical location driven by distal tumors. Differential abundance testing identified tumor-associated genera, and linear discriminant modeling highlighted taxa with high discriminatory power, including *Bacteroides*, *Eubacterium*, *Fusobacterium*, and *Acinetobacter*. Methylome ordination separated tumor from non-tumor samples and revealed prominent contributions of zinc finger (ZNF) loci. Integrative latent-variable modeling (PLS/sparse partial least squares and multi-block sparse partial least squares discriminant analysis) supported coordinated microbiome–methylome variation distinguishing tumor from non-tumor mucosa, prioritized a limited set of bacterial and methylation signals, and suggested partial discrimination between conventional and serrated tumor profiles, particularly in the methylome block. Focusing on *Fusobacterium*, methylation changes in selected host loci were associated with its abundance, and qPCR-based quantification of *Fusobacterium nucleatum* correlated with CpG methylation at ZNF788. *F. nucleatum* levels were also associated with serrated/microsatellite instability-related CRC features and more advanced disease. These findings identify subtype-aware microbiome–methylome signatures in CRC and highlight ZNF788 methylation as a candidate epigenetic correlate of intratumoral *F. nucleatum* burden.

**IMPORTANCE:**

Colorectal cancer does not develop in a single way, and different tumor types may interact differently with bacteria living on the bowel lining. This study examined both tissue-associated bacteria and DNA methylation, an epigenetic mark that helps regulate genes, in paired tumor and nearby non-tumor tissue from patients with colorectal cancer. By analyzing these two layers together, we identified microbial and host methylation patterns linked to tumor tissue and to serrated-pathway cancers. In particular, *Fusobacterium nucleatum* was associated with serrated/microsatellite instability-related features, more advanced disease, and methylation of the host gene *ZNF788*. These findings suggest that combining microbiome and epigenetic information may help explain why colorectal cancer subtypes behave differently and may support future subtype-aware biomarkers.

## INTRODUCTION

Colorectal cancer (CRC) is one of the most frequently diagnosed malignancies and a leading cause of cancer-related deaths worldwide (1–3). Beyond its clinical burden, CRC is biologically heterogeneous; tumors arise through distinct routes to malignancy that differ in histopathology, molecular alterations, anatomical distribution, and clinical behavior (4–7). The conventional adenoma–carcinoma sequence is characterized by stepwise genetic changes and chromosomal instability, as captured by foundational models of colorectal tumorigenesis (5, 6). An alternative serrated pathway is increasingly recognized and encompasses precursor lesions and carcinomas with characteristic morphology and molecular features (8–11). Serrated adenocarcinoma (SAC) and mismatch repair-deficient tumors are endpoints of the serrated pathological pathway and frequently associated with the CpG island methylator phenotype (CIMP) and microsatellite instability (MSI), underscoring the tight coupling between subtype biology and epigenetic reprogramming (12–17).

Over the last decade, the intestinal microbiota has emerged as a key ecological factor linked to CRC, and multiple conceptual frameworks have been proposed to explain how specific microbes contribute to carcinogenesis (18). These include “alpha-bug” models and “driver–passenger” dynamics, in which a limited number of organisms can initiate or shape a tumor-promoting niche, followed by ecological succession as the microenvironment evolves (19–22). Dysbiosis has been linked to CRC through mechanisms such as chronic mucosal inflammation, production of genotoxins and metabolites that alter epithelial proliferation, disruption of barrier integrity, and modulation of antitumor immune surveillance (19, 20, 23–25). Accordingly, clinical and experimental studies have increasingly focused on identifying reproducible CRC-associated taxa, understanding their functional potential, and clarifying whether microbial signals are shared or subtype-specific across CRC phenotypes (3, 19, 20).

As the colonic mucosa is the interface where host tissues and microbes interact, mucosa-associated microbial communities may be particularly relevant to local epithelial biology. Several studies have reported reproducible tumor-associated shifts in microbial composition, including the enrichment of oral-associated taxa and depletion of commensal anaerobes, both in established tumors and in precursor lesions, such as adenomas (26–29). While stool-based signatures are attractive for non-invasive testing, tissue-associated profiles may better capture bacteria embedded within tumor-adjacent niches, including invasive or biofilm-associated organisms with potential clinical relevance (25, 28). These considerations motivate analyses that use paired tumor (T) and adjacent non-tumor mucosa (NT) to disentangle field effects from tumor-specific signals and connect microbial changes with local host biology.

Among CRC-associated taxa, *Fusobacterium*, particularly *Fusobacterium nucleatum*, has been repeatedly detected in colorectal tumors and proposed as a subtype-associated biomarker candidate (28, 29). Mechanistically, *F. nucleatum* can promote pro-tumor signaling via its FadA adhesin, modulating epithelial pathways such as E-cadherin/β-catenin signaling and supporting a context-dependent role in tumorigenesis (30). *Fusobacterium* has also been linked to the inflammatory and immune features of the tumor microenvironment, suggesting that its abundance may reflect and potentially reinforce host programs relevant to tumor progression (31). Clinically, higher *Fusobacterium*/*F. nucleatum* abundance has been associated with molecular subtypes and outcomes, including links to CIMP-related methylation patterns and serrated-pathway lesions (28, 32, 33), associations with tumor stage and disease severity (34), and patient prognosis (34). These observations support a working model in which *Fusobacterium* is not uniformly enriched across all CRC cases but may show preferential relationships with particular tumor subtypes and host regulatory states.

Epigenetic deregulation is a hallmark of CRC. Aberrant DNA methylation at CpG islands can silence tumor suppressor genes, reshape transcriptional programs, and cooperate with genetic alterations to promote malignant transformation (35, 36). In CRC, widespread promoter hypermethylation underlies the CIMP phenotype and is closely intertwined with serrated neoplasia and microsatellite instability-related tumor biology (8–13, 35). Importantly, host epigenetic states are plastic and can respond to inflammatory and environmental cues, providing a plausible interface through which microbial exposure may influence tumor biology (35, 36). Experimental work has shown that perturbations in the gut microbiota can remodel mucosal methylation patterns and immune responses, supporting the existence of microbiome–epigenome crosstalk in the colon (37, 38).

**Fig 1:**
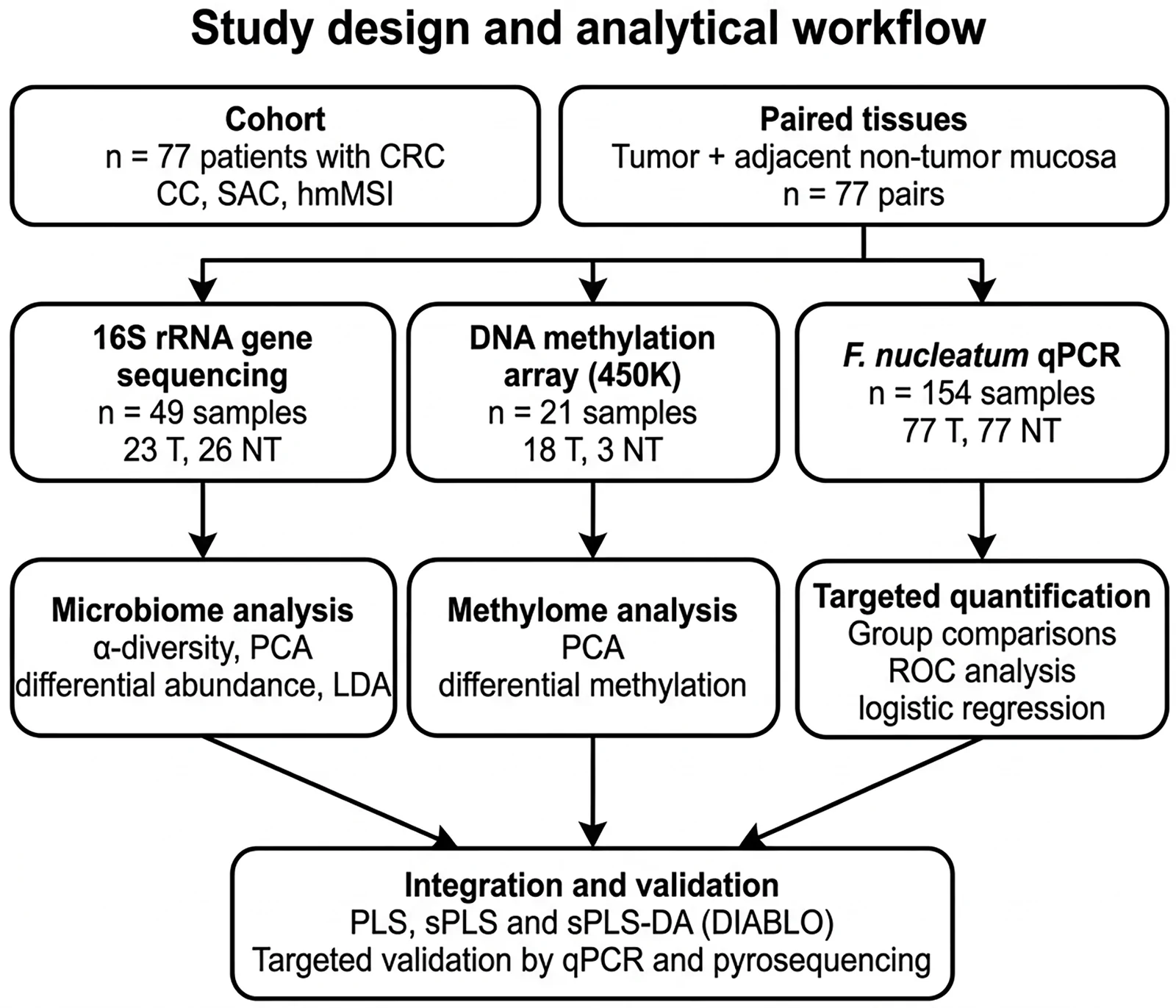
Study design, sample subsets, and analytical workflow. The flow diagram shows the full colorectal cancer cohort, paired tumor (T) and adjacent non-tumor mucosa (NT) samples, and the subsets analyzed by each platform: qPCR (*n* = 154 samples, 77 Ts and 77 NTs), 16S rRNA gene sequencing (*n* = 49 samples, 23 Ts and 26 NTs), and genome-wide DNA methylation profiling using Infinium 450K arrays (*n* = 21 samples, 18 Ts and 3 NTs). The workflow summarizes microbiome profiling, methylome profiling, targeted *F. nucleatum* quantification, integrative modeling using PLS, sPLS, and multi-block sPLS-DA/DIABLO, and targeted validation by qPCR and pyrosequencing. Abbreviations: CC, conventional carcinoma; CRC, colorectal cancer; DIABLO, Data Integration Analysis for Biomarker Discovery Using Latent Variable Approaches for Omics Studies; hmMSI, CRC showing histological and molecular features of microsatellite instability; LDA, linear discriminant analysis; NT, non-tumor mucosa; PCA, principal component analysis; PLS, partial least squares; qPCR, quantitative polymerase chain reaction; SAC, serrated adenocarcinoma; sPLS, sparse partial least squares; sPLS-DA, sparse partial least squares discriminant analysis; T, tumor.

Despite rapid progress in CRC microbiome and epigenome research, integrative analyses that connect mucosa-associated microbial signatures with genome-wide host DNA methylation in matched tumor and adjacent non-tumor tissues remain limited, particularly across clinically meaningful CRC subtypes (19, 20, 39). This gap is relevant because serrated/MSI-related tumors display distinctive methylation programs, and several CRC-associated bacteria appear enriched in specific molecular contexts rather than across CRC as a whole (8–14, 28–34). Here, we combined mucosal 16S ribosomal RNA (rRNA) gene sequencing with CpG methylome profiling in paired tumor and peripheral non-tumor colorectal mucosa from CRC patients stratified by histological and molecular features. Using an integrative modeling framework, we aimed to identify coordinated microbiome–methylome patterns distinguishing tumor from non-tumor mucosa and to evaluate whether *Fusobacterium*/*F. nucleatum* abundance is associated with subtype-defining clinicopathological and molecular features (Fig. 1).

## RESULTS

### Patient cohort and molecular features

The study included 77 patients with paired T and adjacent NT samples. Clinicopathological and molecular characteristics of the cohort are summarized in Table 1. Tumors were classified as conventional CRC (CC, *n* = 31) or serrated-pathway CRC (*n* = 46), including cases diagnosed as SAC and a subgroup of CRC showing histological and molecular features of MSI (hmMSI; *n* = 11). Although serrated tumors are less frequent in the general population than conventional ones, the cohort was intentionally enriched for serrated cases to facilitate comparative analyses between subtypes. Mean age did not differ between CC and SAC (72.57 vs 70.0 years, *P* = 0.237) or between serrated subgroups (SAC vs hmMSI, 70.0 vs 72.8 years; *P* = 0.198). Sex distribution and tumor location were comparable between the CC and SAC (*P* = 0.884 and *P* = 0.244, respectively) and within the serrated subgroups (*P* = 0.396 and *P* = 0.446, respectively). The overall stage distribution was similar between the CC and SAC groups (*P* = 0.834) but differed between the serrated subgroups (*P* = 0.032), with hmMSI cases tending to cluster in more advanced stages than SAC cases.

**TABLE 1:** Clinicopathological and molecular characteristics of the colorectal cancer cohort by subtype^*a*^^*,*^^*b*^

Characteristic	CC (*n* = 31)	SAC (*n* = 46)	hmMSI (*n* = 11)	P*	P**
Age, mean (95% CI)	72.57 (69.1–76.0)	70.0 (67.2–72.7)	72.8 (65.3–80.4)	0.237	0.198
Sex: male	17/31 (54.8%)	26/46 (56.5%)	5/11 (45.5%)	0.884	0.396
Sex: female	14/31 (45.2%)	20/46 (43.5%)	6/11 (54.5%)		
Tumor location: right side	15/31 (48.4%)	29/46 (63.0%)	8/11 (72.7%)	0.244	0.446
Tumor location: left side	16/31 (51.6%)	17/46 (37.0%)	3/11 (27.3%)		
Stage 0–I	5/31 (16.1%)	7/46 (15.2%)	0/11 (0.0%)	0.834	0.032
Stage II (IIA–C)	11/31 (35.5%)	15/46 (32.6%)	4/11 (36.4%)		
Stage III (IIIA–C)	13/31 (41.9%)	18/46 (39.1%)	3/11 (27.3%)		
Stage IV (IVA–B)	2/31 (6.5%)	6/46 (13.0%)	4/11 (36.4%)		
CIMP status (available *N*)	31	38	9	0.040	0.047
CIMP—negative	8/31 (25.8%)	11/38 (28.9%)	4/9 (44.4%)		
CIMP—low	19/31 (61.3%)	13/38 (34.2%)	0/9 (0.0%)		
CIMP—high	4/31 (12.9%)	14/38 (36.8%)	5/9 (55.6%)		
MSI status				<0.001	<0.001
MSS	29/31 (93.5%)	21/46 (45.7%)	0/11 (0.0%)		
MSI-L	2/31 (6.5%)	15/46 (32.6%)	3/11 (27.3%)		
MSI-H	0/31 (0.0%)	10/46 (21.7%)	8/11 (72.7%)		
*BRAF* status (available *N*)	31	38	8	<0.001	0.058
Mutated	0/31 (0.0%)	13/38 (34.2%)	5/8 (62.5%)		
Wild type	31/31 (100.0%)	25/38 (65.8%)	3/8 (37.5%)		
*KRAS* status (available *N*)	30	39	10	0.090	0.021
Mutated	19/30 (63.3%)	16/39 (41.0%)	1/10 (10.0%)		
Wild type	11/30 (36.7%)	23/39 (59.0%)	9/10 (90.0%)		
*PIK3CA* status (available *N*)	28	34	6	0.125	0.216
Mutated	6/28 (21.4%)	2/34 (5.9%)	1/6 (16.7%)		
Wild type	22/28 (78.6%)	32/34 (94.1%)	5/6 (83.3%)		

^*a*^ Values are shown as *n*/*N* (%) unless otherwise indicated. *P* values correspond to CC vs SAC (P*) and SAC vs hmMSI (P**).

^*b*^ The cohort includes paired tumor and adjacent non-tumor mucosa samples from 77 patients. Data are shown as mean ± SD (or median [interquartile range], where indicated) for continuous variables and *n* (%) for categorical variables. *P* values correspond to comparisons between CC and SAC and, where relevant, between serrated subgroups (SAC vs hmMSI) using *χ*^2^/Fisher’s exact test for categorical variables and *t*-test/Mann–Whitney *U* test for continuous variables, as appropriate. Abbreviations: CC, conventional carcinoma; CIMP, CpG island methylator phenotype; hmMSI, CRC showing histological and molecular features of MSI; MSI, microsatellite instability; SAC, serrated adenocarcinoma.

The molecular features were consistent with subtype biology. High-grade CIMP was more frequent in SAC than in CC (14 vs 4, *P* = 0.040). *BRAF* mutations were restricted to serrated tumors (13/38 SAC and 5/8 hmMSI, *P* < 0.001 for CC vs SAC). MSI status differed markedly among the histological groups (*P* < 0.001), with CC cases being predominantly MSS, SAC showing a mixed MSI distribution, and the hmMSI subgroup being strongly enriched in MSI-H tumors. *KRAS* mutations were more frequent in serrated non-MSI tumors than in the hmMSI subgroup (*P* = 0.021), whereas *PIK3CA* mutations did not differ according to the histological group (*P* = 0.125).

Together, these data indicate that the patient cohort was well balanced for major clinical variables while displaying the expected molecular distinctions between conventional and serrated CRC subtypes. The enrichment of serrated cases allows for a focused comparison of subtype-specific molecular and microbiota features, which would not be possible in an unselected population.

### Tissue-associated microbiome profiling

**Fig 2:**
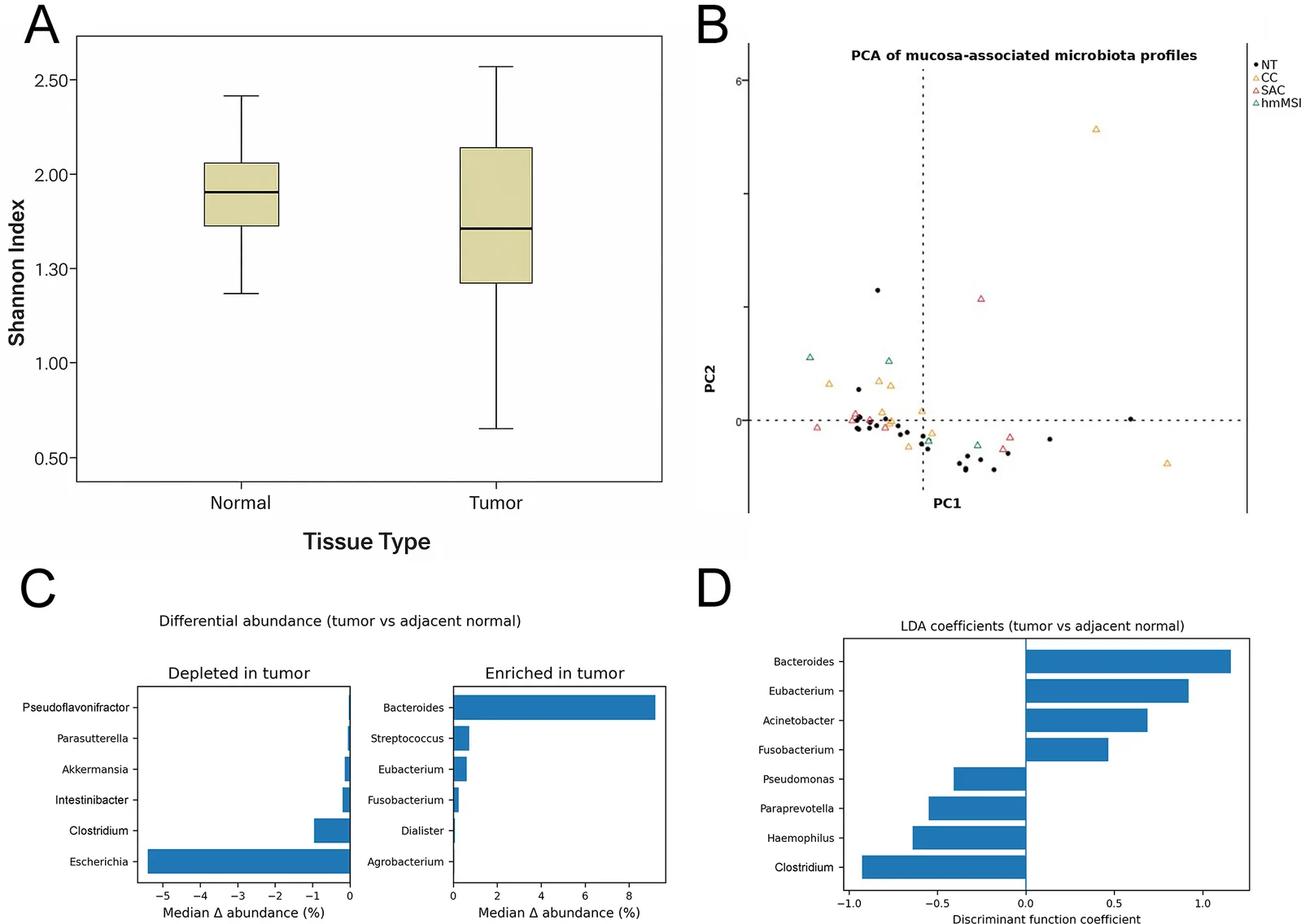
Tumor-associated shifts in mucosa-associated microbiota. (**A**) Alpha diversity, measured using the Shannon index, in tumor (T) and adjacent non-tumor mucosa (NT) samples profiled by 16S rRNA gene sequencing. Boxes show median and interquartile range, and whiskers indicate the data range. *P* value was computed using the Mann–Whitney *U* test. (**B**) Ordination of genus-level microbiota profiles by principal component analysis (PCA). Symbols indicate the sample group: black filled circles, NT; orange triangles, CC; red triangles, SAC; green triangles, hmMSI. Percent variance explained by each component is shown on the axes. (**C**) Differential genus-level abundance between tumor and adjacent non-tumor mucosa, shown as median Δ abundance (%). Positive values indicate enrichment in tumor tissue, whereas negative values indicate depletion in tumor tissue. (**D**) Linear discriminant analysis (LDA) coefficients highlighting the genera contributing most strongly to discrimination of T vs NT samples. Positive coefficients indicate enrichment in T, and negative coefficients indicate enrichment in NT. Full differential abundance results and complete LDA outputs are provided in Tables S1 and S2.

Microbiota composition was assessed by 16S rRNA gene sequencing in a subset of samples with sufficient sequencing output and complete pairing (49 samples: 23 Ts and 26 NTs). Alpha diversity (Shannon index) did not differ between the tumor and adjacent non-tumor mucosa (median [interquartile range] 1.91 [1.65–2.17] vs 2.06 [1.89–2.24], *P* = 0.34) (Fig. 2A). Within the tumor samples, alpha diversity did not differ by histological subtype (Kruskal–Wallis = 0.265, *P* = 0.87).

Exploratory ordination by principal component analysis (PCA) did not show a clear separation between the T and NT samples (Fig. 2B); the first two components explained 17% of the total variance. Univariate differential abundance testing did identify 20 genera with nominal *P* < 0.05 between the T and NT groups (Mann–Whitney *U* test). Genera more abundant in tumors included *Bacteroides* (median difference +9.2%), *Streptococcus* (+0.72%), *Eubacterium* (+0.61%), *Fusobacterium* (+0.25%), *Dialister* (+0.07%), and several low-abundance genera (e.g., *Agrobacterium*, *Delftia*, *Acinetobacter*, and *Stenotrophomonas*). Genera depleted in tumors included *Escherichia* (−5.4%), *Clostridium* (−0.96%), *Akkermansia* (−0.138%), *Parasutterella* (−0.057%), *Pseudoflavonifractor* (−0.033%), and *Intestinibacter*, among others. These percent differences represent the actual change in relative abundance between groups (Fig. 2C). The full genus-level differential abundance results are provided in Table S1.

To prioritize discriminatory taxa, we performed linear discriminant analysis (LDA). In this analysis, positive LDA scores indicate genera more abundant in tumors, while negative scores indicate genera more abundant in adjacent non-tumor mucosa. The magnitude of the score reflects the discriminatory contribution of each genus to the separation of groups, rather than the absolute abundance. LDA identified eight genera with the highest contributions to the separation of T and NT samples (Fig. 2D): *Bacteroides* (coefficient, 1.160), *Eubacterium* (0.920), *Acinetobacter* (0.689), and *Fusobacterium* (0.466) with positive coefficients, and *Clostridium* (−0.927), *Haemophilus* (−0.640), *Paraprevotella* (−0.549), and *Pseudomonas* (−0.408) with negative coefficients. LDA performed between CC and SAC highlighted *Anaerococcus* (31.278), *Phascolarctobacterium* (27.997), *Bacteroides* (25.194), *Actinomyces* (15.509), and *Collinsella* (13.701) among the genera contributing most strongly to histological discrimination (with *Odoribacter*, *Sutterella*, and *Paraprevotella* showing the most negative coefficients). Here again, positive and negative scores reflect enrichment in CC vs SAC, and larger absolute values indicate stronger discriminatory power. Complete LDA outputs for T vs NT and CC vs SAC comparisons are provided in Table S2.

Collectively, these analyses indicate that while overall community structure showed limited differences between tumor and adjacent non-tumor mucosa, a restricted set of bacterial genera displayed consistent tumor-associated shifts and discriminatory potential.

### Methylome profiling highlights tumor-associated hypermethylation of zinc-finger genes

**Fig 3:**
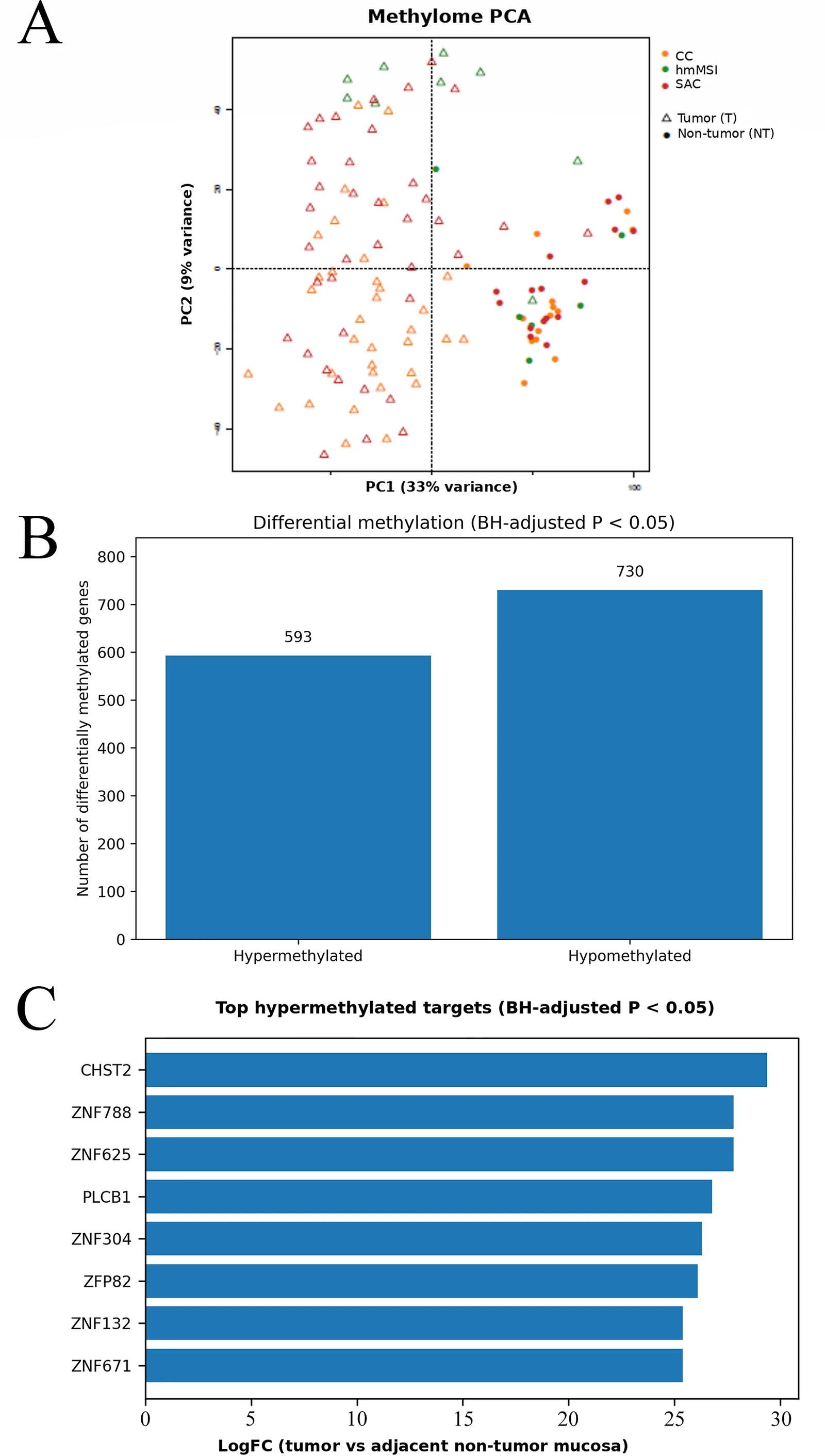
Tumor methylomes separate from adjacent mucosa and highlight prominent ZNF-family signals. (**A**) PCA of genome-wide DNA methylation (Infinium HumanMethylation450), showing separation of tumor (T) and non-tumor mucosa (NT); hmMSI samples are indicated. (**B**) Distribution of differentially methylated genes in tumors, shown as the number of hypermethylated and hypomethylated genes. (**C**) Summary of differential methylation results for tumor vs non-tumor comparisons, with selected highly informative loci highlighted, including ZNF-family genes and CHST2. A summary of the differential methylation results and the top hypermethylated loci is provided in Table S3. Abbreviations: hmMSI, CRC showing histological and molecular features of MSI; ZNF, zinc finger.

Genome-wide DNA methylation profiling was performed in a subset of samples with sufficient DNA and array quality (21 samples: 18 Ts and 3 NTs). PCA of methylation data separated tumor and non-tumor mucosa along PC1 (33% variance), while PC2 (9% variance) captured additional subtype structures, with hmMSI samples clustering apart from CC and SAC (Fig. 3A). Genes contributing strongly to PC1 included multiple zinc-finger loci, such as *ZNF793*, *ZNF625*, *ZNF304*, and *ZNF132,* as well as *CHST2* and *ADAMTS1*.

Differential methylation analysis across 18,569 genes identified 1,323 genes with significant methylation differences between T and NT, including 593 hypermethylated and 730 hypomethylated genes in tumors (Fig. 3B). Among the most strongly hypermethylated genes in tumors, zinc-finger family members were prominent, including ZNF671 (log_2_ fold change [LogFC] 2.54, adjusted *P* = 0.015), ZNF625 (LogFC 2.78, adjusted *P* = 0.014), ZNF788 (LogFC 2.78, adjusted *P* = 0.023), and CHST2 (LogFC 2.94, adjusted *P* = 0.014) (Fig. 3C). A summary of the differential methylation results and the top hypermethylated loci is provided in Table S3.

These analyses identify zinc-finger genes as prominent targets of tumor-associated hypermethylation, providing a focused epigenetic framework for subsequent integrative microbiome–methylome analyses.

### Integrated microbiome–methylome modeling identifies coordinated tumor-associated features

**Fig 4:**
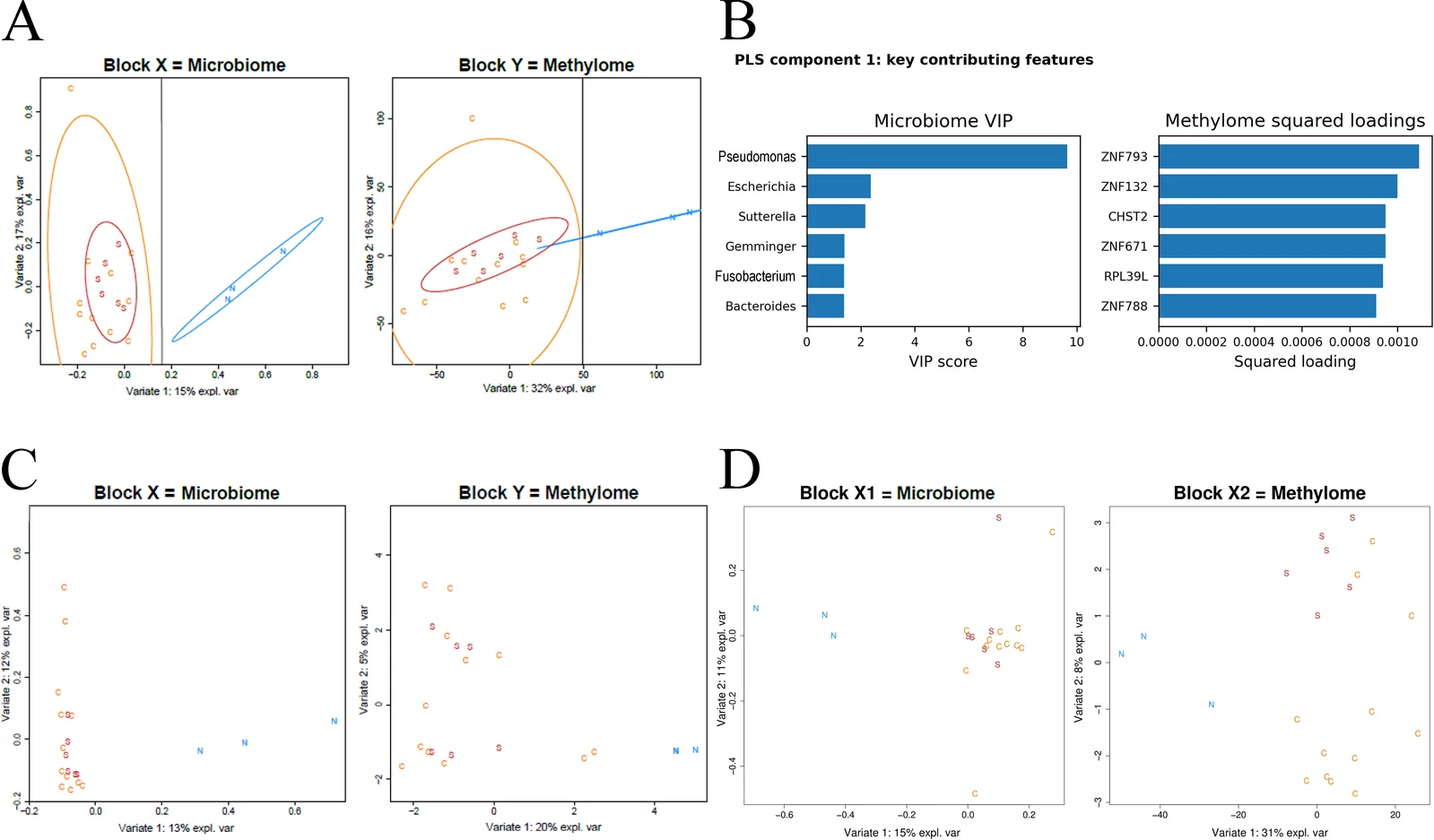
Integrative modeling identifies coordinated microbiome–methylome features associated with tumor status and histological subtype. (**A**) PLS scores plot showing separation of tumor and adjacent non-tumor mucosa along latent variables. (**B**) VIP scores in the microbiome layer and squared loadings in the methylome layer, highlighting features contributing most strongly to the PLS model. (**C**) sPLS cross-omic feature selection summarized as a block plot connecting selected microbial and methylation features, including ZNF788, CHST2, and ZNF671. (**D**) Multi-block sPLS-DA score plot generated within the DIABLO framework, showing separation across microbiome and methylome blocks. Latent variable 1 separates tumor from adjacent non-tumor mucosa, whereas latent variable 2 contributes to partial discrimination between conventional carcinoma and serrated adenocarcinoma, particularly in the methylome block. Full VIP/loading outputs and additional DIABLO visualizations are provided in Table S4 and Fig. S1 and S2. Abbreviations: PLS, partial least squares; sPLS, sparse partial least squares; sPLS-DA, sparse partial least squares discriminant analysis; VIP, variable importance in projection.

To explore the coordinated variation between microbial taxa and host methylation patterns, partial least squares (PLS) modeling was applied to matched microbiome and methylome profiles (*n* = 21, 18 tumor and 3 adjacent non-tumor mucosa samples). Latent variable 1 separated tumor from non-tumor samples in both data layers, whereas PLS did not indicate consistent separation according to histological subtype (Fig. 4A). Variable importance in projection (VIP > 1) indicated that only a small number of microbial genera contributed substantially to the PLS model, dominated by *Pseudomonas* (VIP 9.64) and followed by *Escherichia* (2.36), *Sutterella* (2.17), *Gemmiger* (1.39), *Bacteroides* (1.38), and *Fusobacterium* (1.38). Importantly, the direction of the PLS weights indicated whether each genus contributed to the tumor-associated or non-tumor-associated side of latent variable 1 (Fig. 4B). In the methylome layer, the strongest squared loadings included *ZNF793*, *ZNF132*, *ZNF671*, *CHST2*, *RPL39L*, and *ZNF788*, with loadings likewise indicating whether each locus was associated with the tumor or non-tumor side of latent variable 1 (Fig. 4B).

Sparse partial least squares (sPLS) was then used for feature selection to derive a parsimonious model while preserving discrimination between tumor and non-tumor samples. The reduced model selected *Pseudomonas* (latent variable 1) and *Escherichia* (latent variable 2) as key microbial features and *ZNF788*, *CHST2*, and *ZNF671* (latent variable 1) and *B3GALNT1* (latent variable 2) as key methylation features (Fig. 4C).

We further evaluated subtype discrimination using a multi-block sparse partial least squares discriminant analysis (sPLS-DA) model within the DIABLO framework. This model showed a strong correlation between the microbiome and methylome components (Pearson’s *r* = 0.90). Latent variable 1 separated tumor and adjacent non-tumor samples, whereas latent variable 2 contributed to the discrimination of CC and SAC tumor samples, particularly in the methylome block. Although separation by histological subtype was not complete, the model suggested that a subset of CC cases shared multi-omic profiles more similar to SAC than to the remaining CC samples (Fig. 4D), with selected cross-omic associations visualized by heatmap and circosplot representations (Fig. S1 and S2).

Together, PLS, sPLS, and sPLS-DA prioritized a limited set of cross-omic features distinguishing tumor from adjacent non-tumor mucosa and provided evidence of partial discrimination between conventional and serrated tumor profiles. Full PLS VIP values, methylome squared loadings, sPLS-selected features, sPLS-DA performance metrics, and PLS1 gene–genus associations are provided in Table S4.

### *Fusobacterium*–methylome associations and targeted validation

Given the tumor enrichment of *Fusobacterium*, we assessed the relationship between its abundance (genus level) and CpG island methylation. Correlation analysis identified loci with strong positive (e.g., *RELA* [*r* = 0.84], *DTYMK* [*r* = 0.83], *RAB11FIP5* [*r* = 0.83], and *MTOR* [*r* = 0.80]) and strong negative (e.g., *EDAR* [*r* = −0.77], *C2orf76* [*r* = −0.74], *CCDC14* [*r* = −0.73], and *ZFP64* [*r* = −0.73]) associations. Intersecting correlation results with differentially methylated genes yielded four loci with negative correlations and significant tumor–non-tumor differential methylation (*CILP2*, *SYCP2L*, *DRD1*, and *PTPLAD2*; correlation coefficients −0.505 to −0.609; differential methylation adjusted *P* ≤ 0.048) and five loci with positive correlations and significant differential methylation (*EGFR*, *PLCH1*, *COBL*, *mirLET7D*, and *SFRP5*; correlation coefficients 0.281–0.299; differential methylation adjusted *P* ≤ 0.046). Detailed *Fusobacterium*–methylome correlation results and targeted validation outputs are provided in Table S5.

**Fig 5:**
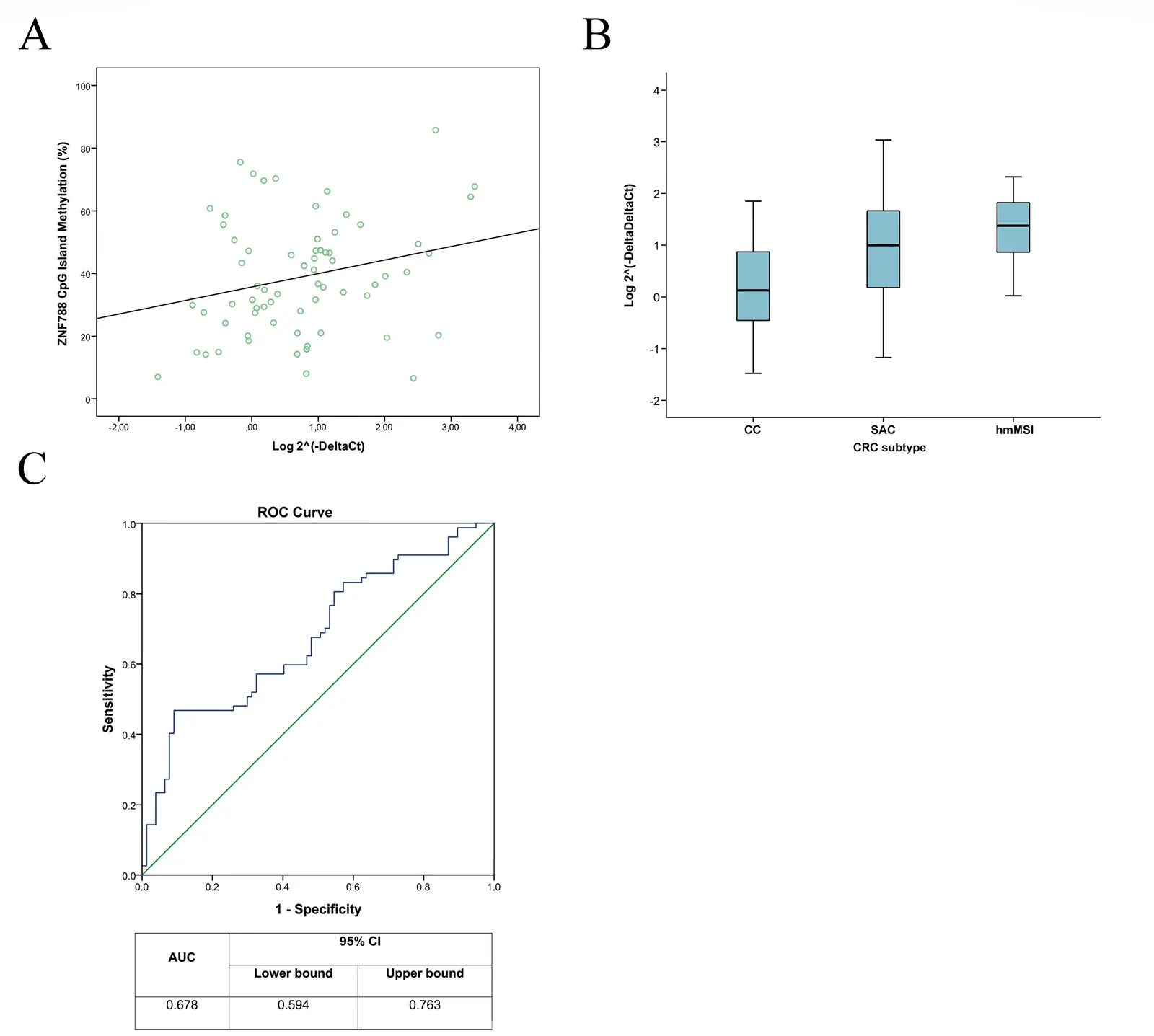
Tumor-associated *Fusobacterium nucleatum* burden associates with a host methylation marker and serrated-related biology. (**A**) Association between *ZNF788* methylation percentage and relative *F. nucleatum* quantification, with Pearson correlation coefficient and *P* value indicated. (**B**) Distribution of tumor *F. nucleatum* levels by CRC subtype (CC, SAC, and hmMSI). (**C**) ROC curve evaluating relative *F. nucleatum* quantification as a discriminator of tumor vs adjacent mucosa, with AUC and 95% CI shown. CC, conventional carcinoma; CRC, colorectal cancer; hmMSI, CRC showing histological and molecular features of microsatellite instability; ROC, receiver operating characteristic; SAC, serrated adenocarcinoma.

Targeted bisulfite pyrosequencing of available samples confirmed tumor–non-tumor methylation differences for several candidates, including *PLCH1* (median 75% vs 56%, *P* < 0.001), *mirLET7D* (83% vs 78%, *P* = 0.001), *EGFR* (43% vs 40%, *P* = 0.040), and *COBL* (77% vs 70%, *P* = 0.002). Next, we tested whether methylation at selected loci tracked with qPCR-based relative quantification of *F. nucleatum*. Among the tested loci, only *ZNF788* methylation showed a significant linear association with relative *F. nucleatum* levels (Pearson’s *r* = 0.278, *F* = 5.179; *P* = 0.026) (Fig. 5A). In the multi-variable linear regression for *ZNF788* methylation percentage, CIMP status (*β* = 14.22, *P* = 0.016) and relative *F. nucleatum* quantification (β = 4.22, *P* = 0.048) were retained, while MSI status showed borderline significance (β = −12.19, *P* = 0.051). Age, tumor stage, location, and sex were excluded from the regression model.

These analyses identify *ZNF788* methylation as a specific epigenetic feature associated with *F. nucleatum* abundance in CRC, independent of major clinicopathological variables.

### qPCR quantification of *F. nucleatum* links tumor-associated burden to subtype, stage, and molecular markers

*F. nucleatum* was quantified by qPCR in the full paired cohort (77 T and 77 NT samples) and normalized to total bacterial load. A high *F. nucleatum* signal (ΔCt ≤ 10 cycles between *F. nucleatum* and total bacteria) was more frequent in tumors than in adjacent non-tumor mucosa (40% [31/77] vs 9% [7/77]), and non-detectable levels were less frequent in tumors (17% [13/77] vs 35% [27/77]). When expressed as normalized relative quantification (2^−ΔΔCt^), 74% (57/77) of cases showed higher tumor-associated *F. nucleatum* levels than in paired NT samples. Because the distribution was strongly right-skewed (skewness coefficient = 5.2), subsequent analyses used log-transformed values.

Log-transformed relative quantification did not differ according to sex (*P* = 0.94) or anatomical location (*P* = 0.57). In contrast, the levels differed by CRC subtype (ANOVA *F* = 5.37, *P* = 0.007), with higher values in serrated-pathway tumors (SAC and hmMSI) than in CC (CC vs SAC, *P* = 0.011; CC vs hmMSI, *P* = 0.002; SAC vs hmMSI, *P* = 0.426) (Fig. 5B). *F. nucleatum* levels also varied with local invasion category (T stage, ANOVA *F* = 14.16; *P* = 0.0001), with significant pairwise differences between early tumors (Tis/T1/T2) and T3 (*P* = 2.1 × 10^−5^), early tumors and T4 (*P* = 0.035), and between T3 and T4 (*P* = 0.043). Across all CRC stages (0–I, II, III, and IV), early stages (0–I) showed lower values than stage II (*P* = 0.00001), stage III (*P* = 0.00004), and stage IV (*P* = 0.03), with an additional difference between stages II and IV (*P* = 0.04).

Associations with molecular markers supported the enrichment of *F. nucleatum* in serrated/MSI-related biology. Relative quantification differed across MSI categories, with higher values in MSI-H than in MSS (*P* = 0.03; MSS vs MSI-L, *P* = 0.26; MSI-L vs MSI-H, *P* = 0.37). *F. nucleatum* levels also differed by *KRAS* and *BRAF* status (*P* = 0.042 and *P* = 0.001, respectively) and were higher in CIMP-high tumors compared with CIMP-low tumors (*P* = 0.027), with a borderline difference vs CIMP-negative cases (*P* = 0.052). No association was observed between *PIK3CA* status and clinicopathological features (*P* = 0.19). Pairwise clinicopathological and molecular associations with log-transformed relative *F. nucleatum* quantification are summarized in Table S6.

Collectively, these data link tumor-associated *F. nucleatum* burden to serrated/MSI-related CRC biology and to markers of tumor progression.

### Diagnostic performance and predictive modeling using *F. nucleatum* quantification

Receiver operating characteristic (ROC) analysis evaluating relative *F. nucleatum* quantification as a discriminator of tumor vs adjacent non-tumor mucosa yielded an AUC of 0.678 (95% CI 0.594–0.763) (Fig. 5C). The Youden-optimized cutoff corresponded to a ΔCt of 10.2 cycles between the *F. nucleatum* and total bacteria qPCRs, achieving 47% sensitivity and 91% specificity (overall accuracy 69%), under the criteria of Ct <40 for *F. nucleatum* qPCR and total bacterial Ct 25 ± 3. In logistic regression modeling for CRC detection, including age and sex, relative *F. nucleatum* quantification was associated with CRC (odds ratio [OR] 2.23, 95% CI 1.51–3.30; *P* = 0.0001), whereas age (*P* = 0.318) and sex (*P* = 0.826) were not significant covariates. In a model predicting serrated vs conventional histology with age and sex as covariates, relative *F. nucleatum* quantification was associated with serrated histology (OR 2.00, 95% CI 1.22–3.28; *P* = 0.006). Extending this analysis to additional covariates identified relative *F. nucleatum* quantification (OR 5.91, 95% CI 1.94–18.03; *P* = 0.002), tumor location (OR 0.19, *P* = 0.049), *KRAS* status (OR 7.10, *P* = 0.043), and *PIK3CA* status (OR 0.01, *P* = 0.013) as variables retained in the best-fitting model. In multi-variable modeling of serrated vs conventional histology, relative *F. nucleatum* quantification remained associated with serrated histology after adjusting for tumor location (right/left sidedness) and mutation status variables retained by model selection. The main logistic regression models are summarized in Table 2, and extended ROC performance and logistic regression outputs are provided in Table S7.

**TABLE 2:** Logistic regression models linking *Fusobacterium nucleatum* quantification with tumor status and serrated histology*^a^*^*,**b*^

Predictor	OR	95% CI	*P*
Model 1: CRC detection (tumor vs adjacent mucosa), adjusted for age and sex			
Relative *F. nucleatum* quantification	2.23	1.51–3.30	0.0001
Age	0.99	0.95–1.02	0.318
Sex	1.08	0.55–2.13	0.826
Model 2: serrated vs conventional histology (SAC vs CC), adjusted for age and sex			
Relative *F. nucleatum* quantification	2.00	1.22–3.28	0.006
Age	0.98	0.94–1.03	0.48
Sex	0.89	0.33–2.36	0.81
Model 3: best-fitting multi-variable model for serrated vs conventional histology			
Relative *F. nucleatum* quantification	5.91	1.94–18.03	0.002
Tumor location	0.19	0.04–0.99	0.049
*KRAS* status	7.10	1.06–47.54	0.043
*PIK3CA* status	0.01	<0.001–0.35	0.013

^*a*^ ROC analysis for tumor vs adjacent mucosa: AUC 0.678 (95% CI, 0.594–0.763). ORs are reported with 95% confidence intervals. Tumor location was coded as right sided vs left sided.

^*b*^ ORs, 95% CIs, and *P* values are reported for qPCR-based relative *F. nucleatum* quantification (log transformed, where indicated) and covariates. Model 1 evaluates CRC detection (tumor vs adjacent mucosa) adjusting for age and sex. Model 2 evaluates serrated vs conventional histology adjusting for age and sex. The multi-variable “best-fitting” model includes variables retained after model selection and reports OR (95% CI) and *P* values for each retained predictor. Abbreviations: CI, confidence interval; CRC, colorectal cancer; OR, odds ratio.

Our results indicate that *F. nucleatum* quantification could provide moderate diagnostic discrimination and was independently associated with serrated CRC biology within multi-variable models.

## DISCUSSION

In this study, we combined tissue-associated microbiome profiling with host DNA methylation analysis to investigate coordinated host–microbe patterns in CRC across conventional and serrated-related tumor subtypes. We hypothesized that long-standing host–microbiota coexistence has shaped human gene regulation, in part through microbe-linked modulation of DNA methylation programs, and that modern dysbiosis driven by lifestyle factors and host genetic susceptibility may shift these epigenetic states toward more pro-carcinogenic phenotypes. Three main observations were made. First, although overall alpha-diversity differences between tumor and adjacent non-tumor mucosa were modest, genus-level shifts and discriminatory modeling highlighted a limited set of taxa that consistently distinguished tumor from non-tumor tissue. Second, tumor methylomes were clearly separated from non-tumor mucosa and were characterized by extensive differential methylation, with a prominent contribution of zinc finger (ZNF) loci among the most informative features. Third, integrative modeling supported coordinated microbiome–methylome variation associated with tumor status and prioritized a small subset of microbial and methylation features, among which *Fusobacterium*/*F. nucleatum* emerged as clinically and molecularly relevant, particularly in serrated/MSI-related contexts.

### Tissue-associated dysbiosis and tumor-adjacent non-tumor mucosa comparisons

Many CRC microbiome studies have reported dysbiosis signatures; however, heterogeneity in specimen type and confounding by stool vs mucosal sampling remain major challenges. By focusing on paired tumors and adjacent non-tumor mucosa, our data emphasize that tumor-associated differences are more apparent at the level of specific taxa than in global diversity metrics. This is consistent with models in which CRC-associated bacteria are not simply a function of overall community richness but rather reflect ecological selection within tumor-adjacent niches (19–22). The genera prioritized by LDA included taxa frequently reported in CRC studies (e.g., *Bacteroides*, *Eubacterium*, and *Fusobacterium*) and a set of genera that may represent niche opportunists, low-abundance markers, or local contaminants introduced during sampling/processing. Importantly, the paired design helps mitigate inter-individual variability but does not eliminate “field effects,” as adjacent non-tumor mucosa in CRC patients may already reflect systemic or local tumor-driven changes in host physiology and microbial colonization.

### Host DNA methylation patterns and subtype biology

Methylome ordination separated tumor from non-tumor tissue and revealed widespread differential methylation patterns, consistent with the established role of epigenetic disruption in CRC and the serrated pathway (8–14, 35, 36). The enrichment of ZNF loci among the top contributors was notable. ZNF family genes, many of which encode transcriptional regulators, are frequently affected by epigenetic silencing in cancer and may serve as sensitive reporters of tumor-associated methylation programs. The observation that hmMSI samples displayed distinct methylome clustering aligns with the known coupling between MSI, CIMP-high patterns, and serrated-related tumor biology (8–14). Together, these methylation signals provide an epigenetic framework for interpreting potential microbial associations.

### Integrated modeling prioritizes a small set of coordinated features

A central goal of this study was to move beyond separate lists of “differential taxa” and “differentially methylated genes” by applying an integrative framework. PLS and sparse feature selection by sPLS prioritized a limited set of bacterial and methylation features associated with tumor status, whereas supervised multi-block sPLS-DA suggested partial discrimination between conventional and serrated tumor profiles. This subtype-related separation was not complete, but it was more apparent in the methylome block, consistent with the central role of DNA methylation in the serrated/MSI-related route to CRC. In the microbiome layer, *Pseudomonas* and *Escherichia* were prominent in VIP and sparse feature selection, whereas in the methylome layer, ZNF genes (including ZNF788), CHST2, ZNF671, and B3GALNT1 were prioritized. These findings illustrate a recurring challenge in multi-omic integration: features that best separate samples in latent-variable models may not always correspond to the most immediately interpretable biological candidates, particularly when working with small sample sizes or compositional data. Nonetheless, the convergence of ZNF family methylation features provides a candidate axis—transcriptional regulation and epigenetic control—that is consistent with CRC subtype biology and warrants further validation.

Several taxa other than *Fusobacterium* also emerged from the differential abundance, LDA, and integrative analyses, including *Bacteroides*, *Eubacterium*, *Pseudomonas*, *Escherichia*, and *Sutterella*. These organisms may reflect tumor-associated ecological shifts, subtype-related microbial niches, or technical and compositional effects inherent to tissue-associated microbiome profiling, and they deserve further investigation in larger studies. Our decision to prioritize *Fusobacterium*/*F. nucleatum* for targeted downstream analyses was therefore based not solely on its rank or effect size in the present data set but on the convergence between our observations and extensive prior biological and clinical evidence linking *F. nucleatum* to CRC biology.

### *F. nucleatum* links microbial burden to epigenetic and molecular phenotypes

Among the candidate bacteria, *Fusobacterium*/*F. nucleatum* is one of the most consistently implicated taxa in CRC and has been associated with inflammatory signaling, immune modulation, and tumor progression in experimental and clinical studies (28–34). Importantly, evidence also supports a functional contribution of intratumoral *F. nucleatum* to disease progression and therapeutic opportunities, including its persistence in colorectal cancer liver metastases and the reduction of *Fusobacterium* load and tumor growth after metronidazole treatment in patient-derived xenograft models (40). Based on these observations, we focused our downstream analyses on host methylation changes associated with *F. nucleatum* presence and burden in human CRC tissues. In our cohort, qPCR-based quantification showed that tumor tissue frequently carried a higher *F. nucleatum* burden than adjacent non-tumor mucosa and that higher levels were associated with serrated-pathway CRC, advanced invasion and stage, and MSI/CIMP and oncogenic alterations. Previous evidence has linked *F. nucleatum* to adenoma subsets with serrated histology, but its association with endpoints of the serrated pathway, such as SAC and hmMSI, had not been reported (31, 34, 41). All these findings are consistent with prior reports linking *F. nucleatum* to specific molecular subtypes and clinical outcomes (31–34), supporting the idea that bacterial enrichment is context dependent rather than uniform across CRC cases.

A key translational observation is the association between *F. nucleatum* levels and methylation of *ZNF788*. However, this association was modest in magnitude despite reaching statistical significance and should therefore be interpreted as hypothesis generating. The result supports *ZNF788* methylation as a candidate epigenetic correlate of intratumoral *F. nucleatum* burden, but it does not establish a causal relationship. Nevertheless, this relationship is consistent with the broader concept of microbiome–epigenome crosstalk, in which microbial exposure or microbially driven inflammation may influence host epigenetic states (35–37). One plausible interpretation is that *F. nucleatum* colonization is facilitated by or contributes to a local tissue environment characterized by epigenetic reprogramming and subtype-specific host responses. Alternatively, both *F. nucleatum* burden and *ZNF788* methylation may be downstream readouts of the same upstream process, such as immune infiltration, mucosal barrier changes, or the metabolic milieu, particularly in serrated/MSI-related tumors. Discriminating between these possibilities will require longitudinal sampling and the use of mechanistic models.

### Biomarker implications

We evaluated the diagnostic potential of *F. nucleatum* quantification and observed moderate discrimination between tumors and adjacent non-tumor mucosa. Although the AUC and sensitivity/specificity estimates support an association with tumor presence, they also highlight that *F. nucleatum* alone is unlikely to serve as a universal, stand-alone marker for CRC across all contexts. Instead, our data support its use as part of a multi-marker strategy tailored to CRC heterogeneity, particularly for serrated-related and CRC showing histological and molecular features of MSI (16, 17). Integrative approaches that combine microbial burden with subtype-relevant methylation features may yield more robust classifiers than either layer alone, especially if optimized in larger cohorts and validated in independent materials.

### Limitations

This study had several limitations. Microbiome sequencing and methylome profiling were performed in subsets of the full paired cohort, and the methylome non-tumor group was small, which constrained power and increased uncertainty in feature selection and integrative modeling. Tissue-associated microbiome profiling using 16S rRNA sequencing provides genus-level resolution and relative abundance; however, it does not resolve strain-level differences or directly capture functional genes. As with all cross-sectional human studies, causal direction cannot be inferred, and residual confounding by diet, medication exposure, bowel preparation, or unmeasured clinical factors may contribute to the observed associations. Finally, “adjacent non-tumor mucosa” may already exhibit field effects and therefore may not reflect a truly healthy baseline. Although PLS, sPLS, and multi-block sPLS-DA are appropriate for exploratory integration of high-dimensional microbiome and methylome data, these models were fitted in a limited subset of samples. Therefore, individual selected features should not be interpreted as definitive biomarkers but rather as candidates emerging from an exploratory framework. The small number of non-tumor methylome samples is particularly relevant because it may influence latent-variable structure, feature-selection stability, and the generalizability of cross-omic associations.

### Conclusions

Our results support a model in which CRC tumor status is associated with coordinated shifts in tissue-associated bacterial communities and host DNA methylation patterns, and in which *F. nucleatum* abundance relates to endpoint CRCs from the serrated pathway and to candidate methylation features, including *ZNF788*. This study provides a framework for multi-omic integration in CRC using paired mucosal tissues and highlights candidate microbial–epigenetic axes for validation and mechanistic exploration. Future studies should prioritize larger, subtype-stratified cohorts, expand to metagenomic and transcriptomic layers, and incorporate longitudinal sampling to test whether microbial–epigenetic signatures precede tumor development or reflect tumor-driven ecological selection within the colonic niche.

## MATERIALS AND METHODS

### Study design, patients, and clinicopathological classification

This study analyzed paired colorectal T and NT specimens from 77 patients diagnosed with CRC who underwent surgical resection between 2004 and 2016 at Hospital General Universitario Santa Lucía (Cartagena, Spain). Patients were included if they were adults (≥18 years), provided written informed consent, and had no concomitant intestinal diseases. The exclusion criterion was antibiotic exposure within the previous three months. Clinicopathological variables (age, sex, tumor location, and TNM stage) were recorded. Tumors were classified by histopathological review according to established morphological criteria into CC and serrated-pathway carcinomas, including SAC. CRCs fulfilling histological and molecular features of MSI were designated hmMSI (42).

### Sample collection and storage

Paired T and NT tissue samples were collected from the surgical specimens. Biopsies (approximately 20–30 mg) were embedded in optimal cutting temperature compound (OCT) and stored at −80°C until DNA extraction.

### DNA extraction from tissue

Genomic DNA was extracted from 5 to 15 mg of OCT-preserved tissues. Samples were homogenized using a TissueRuptor (QIAGEN, Hilden, Germany), incubated in ATL buffer with proteinase K at 56°C for 3 h, and processed using the QIAamp DNA Mini Kit (QIAGEN) on the QIAcube automated platform, following the manufacturer’s instructions. DNA was quantified using spectrophotometry (NanoDrop; Thermo Fisher Scientific, Waltham, MA, USA) and stored at −80°C until downstream applications.

### 16S rRNA gene amplicon library preparation and Illumina MiSeq sequencing

Bacterial community profiling was performed by sequencing the V3–V4 region of the 16S rRNA gene. Amplicon libraries were prepared following the Illumina 16S Metagenomic Sequencing Library Preparation workflow using locus-specific primers containing Illumina overhang adapters (V3–V4: 341F/805R). Briefly, PCR reactions (25 µL) contained KAPA HiFi HotStart ReadyMix, primers (0.2 µM), and template DNA (2.5 µL at 5 ng/µL concentration). The cycling conditions were as follows: 95°C for 3 min; 25 cycles of 95°C for 30 s, 55°C for 30 s, and 72°C for 30 s; and a final extension at 72°C for 5 min. Amplicons were verified by capillary electrophoresis and purified using AMPure XP beads. Indexing was performed using the Nextera XT Index Kit (index PCR, 50 µL), with the following cycling conditions: 95°C for 3 min; 8 cycles of 95°C for 30 s, 55°C for 30 s, and 72°C for 30 s; and 72°C for 5 min. Indexed libraries were purified (AMPure XP), quantified (Qubit 2.0), normalized to 4 nM, and pooled at equimolar concentrations. The final pool was denatured with 0.2 N NaOH, diluted to a final loading concentration of 4 pM, and spiked with PhiX control (≥5%, used at 10% in this study). Paired-end sequencing was performed on an Illumina MiSeq platform using the MiSeq Reagent Kit v3 (600 cycles, 2 × 300 bp). Microbiome profiling for downstream analyses was conducted in a subset of samples for which sequencing output and clinical pairing were available (*n* = 49 total: 23 Ts and 26 NTs), yielding genus-level relative abundance tables.

### DNA methylation profiling by Infinium HumanMethylation450 BeadChip

Genome-wide DNA methylation was assessed in a subset of samples with sufficient DNA using Infinium HumanMethylation450 BeadChip (Illumina). Briefly, bisulfite-converted DNA was hybridized to BeadChips, scanned on the Illumina iScan system, and processed in GenomeStudio (Methylation Module). Methylation levels were expressed as β-values (0–1 scale) after background correction and control-based normalization. For differential methylation, tumor vs non-tumor comparisons were performed using probe-level β-values and gene-level summaries derived from CpG island methylation percentages. A CpG site was considered differentially methylated when it showed a β-value difference of >0.6 with *P* ≤ 0.05. LogFCs were calculated from the mean methylation percentages, and multiple testing correction was applied where indicated. Methylation profiling for microbiome–methylome integration included *n* = 21 samples (18 Ts and 3 NTs).

### Bisulfite conversion and pyrosequencing validation

For targeted validation of the selected loci, DNA was bisulfite converted using the EZ DNA Methylation-Direct Kit (Zymo Research) according to the manufacturer’s protocol (including incubation at 50°C for up to 16 h). Bisulfite-treated DNA was amplified using locus-specific primers (one biotinylated), and methylation was quantified by pyrosequencing on a PyroMark Q24 instrument, using PyroMark Gold Q24 reagents. PCR cycling for pyrosequencing assays was performed at 95°C for 15 min, 45 cycles of 94°C for 30 s, 56°C for 30 s, and 72°C for 30 s, followed by 72°C for 10 min. Primer sequences and assay designs for each validated locus are provided in Table S8.

### Quantification of total bacterial load and *Fusobacterium nucleatum* by qPCR

*F. nucleatum* and total bacterial DNA were quantified by real-time PCR using QuantiTec SYBR Green PCR Master Mix (QIAGEN) on an Applied Biosystems 7500 Fast System. Primer sets targeted (i) total bacteria (16S rRNA), (ii) *F. nucleatum*, and (iii) human β-actin (primer sequences provided in Table S8). The thermal cycling conditions were as follows: 95°C for 10 min, followed by 40 cycles of 95°C for 15 s and 60°C for 1 min, with post-amplification melting-curve analysis. Data were analyzed using SDS software (Applied Biosystems, Foster City, CA, USA). Samples were considered evaluable if the Ct for total bacteria was within the expected range (approximately 25 ± 3 cycles). *F. nucleatum* detection was accepted when Ct was <40 and when the Ct difference between total bacteria and *F. nucleatum* did not exceed 10 cycles. Relative *F. nucleatum* abundance was calculated by the 2^−ΔΔCt^ method, normalizing *F. nucleatum* to total bacterial load. qPCR analyses were performed in the full paired cohort (154 samples from 77 patients, T and NT tissues).

### MSI analysis

MSI status was determined using the MSI Analysis System (Promega), which evaluates five mononucleotide markers (BAT-25, BAT-26, NR-21, NR-24, and MONO-27). Fragment analysis was interpreted according to the manufacturer’s criteria to assign the MSI status.

### CIMP assessment by methylation-specific multiplex ligation-dependent probe amplification

CIMP status was assessed using methylation-specific multiplex ligation-dependent probe amplification (MS-MLPA) with the SALSA MS-MLPA Probemix ME042-C1 (MRC-Holland, Amsterdam, Netherlands). The reactions included parallel digestion with the methylation-sensitive restriction enzyme HhaI and analysis of fragment profiles. Data were processed using Coffalyser.Net software (MRC-Holland), and CIMP categories (CIMP-0, CIMP-L, and CIMP-H) were assigned according to the established probe panel thresholds.

### Somatic mutation analysis (*KRAS*, *BRAF*, and *PIK3CA*)

Somatic mutations were assessed by pyrosequencing using commercial assays (Therascreen Pyro Kit Series, QIAGEN) according to the manufacturer’s protocols. Briefly, the target regions were amplified by PCR and analyzed on the PyroMark Q24 platform (QIAGEN). Mutation calls and variant allele frequencies were assigned using the PyroMark Q24 software (QIAGEN), which calculates the ratio of mutant to wild-type alleles based on the peak heights in the generated pyrograms, comparing them against the assay-specific reference controls.

### Statistical analysis and integrative analyses

Microbiome differences between T and NT samples were assessed using Mann–Whitney *U* tests for genus-level abundance comparisons, followed by LDA to identify discriminatory taxa. PCA was used for exploratory ordination. For integrative microbiome–methylome modeling, PLS, sPLS, and multi-block sPLS-DA were applied to matched microbiome (*X*_1_) and methylome (*X*_2_) matrices. The sPLS-DA model was implemented within the DIABLO framework (mixomics R package) to maximize covariance between omics blocks while evaluating discrimination according to tissue type and histological subtype (*Y*). Correlations between *Fusobacterium* (genus-level relative abundance or *F. nucleatum* qPCR) and DNA methylation metrics were evaluated using Pearson’s correlation in R (stats cor function). Associations between *F. nucleatum* qPCR levels and CRC molecular markers were tested using parametric (*t*-test and ANOVA) or non-parametric (Mann–Whitney and Kruskal–Wallis) tests as appropriate. Multi-variable linear regression was used to control for potential confounding factors, and ROC curve analysis with logistic regression was applied to evaluate the diagnostic performance of *F. nucleatum* quantification. A two-sided *P* value of <0.05 was considered statistically significant. For high-dimensional methylation analyses, *P* values were adjusted for multiple comparisons using the Benjamini–Hochberg false discovery rate procedure. Unless stated otherwise, adjusted *P* values (*q* values) are reported for omics-wide methylation feature lists, whereas nominal *P* values are reported for genus-level differential abundance testing and for targeted validations (qPCR/pyrosequencing). R (v3.4.0, RStudio IDE v1.0.143) was used for correlation analyses, whereas other statistical analyses were performed using SPSS v21.0.

### STORMS reporting checklist

The completed STORMS checklist for reporting this human microbiome study has been deposited in Zenodo and is available at https://doi.org/10.5281/zenodo.21218759.

### Study limitations

We acknowledge several limitations. First, the observational and cross-sectional nature of our design prevents us from inferring causality. Although our data link specific bacterial taxa—particularly *F. nucleatum*—to methylation patterns enriched in serrated/MSI-related CRC, we cannot determine whether these microbes contribute to epigenetic reprogramming or preferentially colonize a tumor microenvironment that is already altered. Resolving this “cause-or-consequence” question will require functional models and/or longitudinal sampling.

Second, because serrated histology is tightly linked to right-sided location and MSI/BRAF status, multi-variable models that include these highly collinear variables can be unstable. We therefore adjusted for tumor location and report associations with key molecular features in complementary analyses rather than interpreting any single model as fully disentangling subtype biology.

Third, integrative multi-omics analyses were performed in a modest subset with limited non-tumor methylome samples, which reduces power and increases uncertainty in feature selection. In addition, tissue-based 16S profiling represents a low-biomass setting and may be sensitive to technical variation. For these reasons, independent validation in larger, subtype-stratified cohorts and external data sets will be essential to assess generalizability and to prioritize candidates for mechanistic follow-up.

## Data Availability

The clinical and multi-omics data sets used in this study are available through ColPortal (https://colportal.imib.es/), an open web platform integrating clinical, histopathological, methylome, microbiome, transcriptome, and microtranscriptome data from colorectal cancer cohorts, as described previously (39). The specific ColPortal sections relevant to this study are the Microbiome section (https://colportal.imib.es/colportal/microbiota.jsf) and the Methylome section (https://colportal.imib.es/colportal/metilacion.jsf). Within ColPortal, readers can access the data through the “Omics” menu and use the clinical-case filters, including tumor status, histological type, MSI status, CIMP status, and Patient ID. ColPortal omics data can be linked to clinical metadata using the field labeled “Id.” The public records corresponding to the data sets are as follows: microbiome analyzed data, https://doi.org/10.6084/m9.figshare.7799129.v2; microbiome raw data, https://doi.org/10.6084/m9.figshare.7799123.v4 microbiome sequencing project, https://identifiers.org/ena.embl:PRJEB31726; methylation raw data, https://doi.org/10.6084/m9.figshare.7782947.v2; serrated carcinoma methylome data set, https://identifiers.org/geo:GSE68060; clinical cases metadata, https://doi.org/10.6084/m9.figshare.7807799.v1; and R scripts used for ColPortal analyses, https://doi.org/10.6084/m9.figshare.7799126.v1. Study-specific processed outputs supporting the present analyses are provided in Tables S1 to S8. Additional analysis files are available from the corresponding authors upon reasonable request, subject to applicable ethical and data protection restrictions.

## SUPPLEMENTAL MATERIAL

Supplemental Tables and Figures msystems.00622-26-s0001.pdf Tables S1-S8 and Figures S1 and S2.

ASM does not own the copyrights to Supplemental Material that may be linked to, or accessed through, an article. The authors have granted ASM a non-exclusive, world-wide license to publish the Supplemental Material files. Please contact the corresponding author directly for reuse.
