# Supplementary material for: Integrative microbiome–methylome analysis links bacterial taxa to colorectal cancer histological subtypes
Source: mSystems. 2026 Aug 24;11(9):e00622-26. doi: 10.1128/msystems.00622-26 (PMC13595989; doi:10.1128/msystems.00622-26)
Supplement: Supplementary file 1 [file msystems.00622-26-s0001.pdf]

Supplementary Tables and Figures

Integrative microbiome-methylome analysis links bacterial taxa to colorectal cancer histological subtypes

Supplementary Table S1. Genus-level differential abundance between tumour and adjacent non-tumour mucosa

Bacterial genus	Median difference (%)	Mann-Whitney-Wilcoxon *p*	Direction
Bacteroides	9.2	0.035	More abundant in T
Streptococcus	0.72	0.005	More abundant in T
Eubacterium	0.61	0.008	More abundant in T
Fusobacterium	0.25	0.012	More abundant in T
Dialister	0.07	0.034	More abundant in T
Agrobacterium	0.05	<0.001	More abundant in T
Delftia	0.04	<0.001	More abundant in T
Acinetobacter	0.02	0.036	More abundant in T
Stenotrophomonas	0.005	0.002	More abundant in T
Acidaminococcus	-0.001	0.002	Less abundant in T
Thermanaerovibrio	-0.003	0.001	Less abundant in T
Butyrivibrio	-0.006	0.010	Less abundant in T
Eisenbergiella	-0.008	0.044	Less abundant in T
Citrobacter	-0.030	0.029	Less abundant in T
Pseudoflavonifractor	-0.033	0.019	Less abundant in T
Parasutterella	-0.057	0.027	Less abundant in T
Akkermansia	-0.138	0.004	Less abundant in T
Intestinibacter	-0.2	0.017	Less abundant in T
Clostridium	-0.96	0.021	Less abundant in T
Escherichia	-5.4	0.030	Less abundant in T

Median difference is reported as tumour minus adjacent non-tumour mucosa. Positive values indicate higher relative abundance in tumour tissue. p values are nominal.

Supplementary Table S2. Linear discriminant analysis (LDA) outputs

S2A. LDA for tumour versus adjacent non-tumour mucosa

Bacterial genus	Discriminant coefficient	Higher in
Bacteroides	1.160	T
Eubacterium	0.920	T
Acinetobacter	0.689	T
Fusobacterium	0.466	T
Pseudomonas	-0.408	NT
Paraprevotella	-0.549	NT
Haemophilus	-0.640	NT
Clostridium	-0.927	NT

S2B. LDA for conventional carcinoma versus serrated adenocarcinoma

Bacterial genus	Discriminant coefficient	Higher in
Anaerococcus	31.278	CC
Phascolarctobacterium	27.997	CC
Bacteroides	25.194	CC
Actinomyces	15.509	CC
Collinsella	13.701	CC
Clostridium	7.217	CC
Parasutterella	2.789	CC
Oribacterium	-1.412	SAC
Holdemanella	-2.788	SAC
Coprobacter	-5.537	SAC
Anaerotruncus	-7.126	SAC
Paraprevotella	-15.020	SAC
Sutterella	-19.674	SAC
Odoribacter	-19.775	SAC

For T versus NT, positive coefficients indicate higher abundance in tumour tissue and negative coefficients indicate higher abundance in adjacent non-tumour mucosa. For CC versus SAC, positive coefficients indicate higher abundance in CC and negative coefficients indicate higher abundance in SAC.

Supplementary Table S3. Differential methylation summary and top hypermethylated loci

S3A. Summary of differential methylation analysis

Metric	Value
Total genes assessed	18,569
Genes with significant methylation differences between T and NT	1,323
Hypermethylated genes in tumours	593
Hypomethylated genes in tumours	730

S3B. Top hypermethylated genes in tumour samples

Gene	LogFC	Adjusted *p* value
ZNF671	2.54	0.015
ZNF132	2.54	0.017
ZFP82	2.61	0.014
ZNF304	2.63	0.031
PLCB1	2.68	0.014
ZNF625	2.78	0.014
ZNF788	2.78	0.023
CHST2	2.94	0.014

LogFC denotes log2 fold-change between tumour and adjacent non-tumour mucosa. Adjusted p values correspond to Benjamini-Hochberg.

Supplementary Table S4. Integrative PLS/sPLS/sPLS-DA feature outputs

S4A. PLS latent variable 1: microbiome VIP values and methylome squared loadings

Microbiome genus	VIP value	Methylome gene	Squared loading
Pseudomonas	9.640962	ZNF793	0.0010939614
Escherichia	2.358396	ZNF132	0.0009982859
Sutterella	2.167052	ZNF671	0.0009543150
Gemmiger	1.393967	CHST2	0.0009483187
Bacteroides	1.385356	RPL39L	0.0009440645
Fusobacterium	1.382742	ZNF788	0.0009101430

S4B. Features selected by the sparse PLS (sPLS) model

Omics block	Latent variable	Selected features
Microbiome	Latent variable 1	Pseudomonas
Microbiome	Latent variable 2	Escherichia
Methylome	Latent variable 1	*ZNF788; CHST2; ZNF671*
Methylome	Latent variable 2	*B3GALNT1*

S4C. Multiblock sPLS-DA model performance by leave-one-out cross-validation

Latent variable	NT error	CT error	ST error	Balanced error rate (BER)
LV1	0	0	1	0.3333333
LV2	0	0.1666667	0.5	0.2222222

S4D. PLS1 associations between selected methylation features and bacterial genera

Methylome gene	Rank	Associated bacterial genus	VIP value
ZNF793	1	*Pseudomonas*	7.88
ZNF793	2	*Escherichia*	3.24
ZNF793	3	*Sutterella*	2.51
ZNF793	4	*Klebsiella*	2.11
ZNF132	1	*Pseudomonas*	9.16
ZNF132	2	*Fusobacterium*	3.39
ZNF132	3	*Escherichia*	2.67
ZNF132	4	*Roseburia*	1.41
ZNF671	1	*Pseudomonas*	9.76
ZNF671	2	*Sutterella*	1.88
ZNF671	3	*Escherichia*	1.81
ZNF671	4	*Gemmiger*	1.51
CHST2	1	*Pseudomonas*	9.70
CHST2	2	*Escherichia*	2.23
CHST2	3	*Klebsiella*	1.61
CHST2	4	*Roseburia*	1.46
RPL39L	1	*Pseudomonas*	6.90
RPL39L	2	*Escherichia*	4.23
RPL39L	3	*Fusobacterium*	3.16
RPL39L	4	*Streptococcus*	3.13
ZNF788	1	*Pseudomonas*	8.84
ZNF788	2	*Escherichia*	2.44
ZNF788	3	*Faecalibacterium*	2.17
ZNF788	4	*Sutterella*	2.00

CT, tumour conventional carcinoma; ST, tumour serrated adenocarcinoma; NT, non-tumour mucosa. S4D lists the four highest VIP genera for each selected methylation feature.

Supplementary Table S5. *Fusobacterium*-methylome associations and targeted validation

S5A. Top methylation correlations with *Fusobacterium spp.*

Positive correlation gene	Pearson r	Negative correlation gene	Pearson r
RELA	0.84	*EDAR*	-0.77
DTYMK	0.83	*C2orf76*	-0.74
RAB11FIP5	0.83	*CCDC14*	-0.73
MTOR	0.80	*ZFP64*	-0.73
PSPH	0.78	*SFMBT1*	-0.73
HIVEP2	0.77	*SESTD1*	-0.72
FAM193B	0.77	*ZBTB39*	-0.72
C20orf112	0.76	*NBPF7*	-0.70
CEP164	0.76	*XCL2*	-0.70
SLC25A13	0.76	*PROCA1*	-0.69

S5B. Negatively correlated differentially methylated genes

Gene	Correlation with *Fusobacterium*	LogFC	Adjusted *p* value
CILP2	-0.505	0.689	0.025
SYCP2L	-0.524	1.251	0.042
DRD1	-0.567	1.572	0.042
PTPLAD2	-0.609	1.809	0.048

S5C. Positively correlated differentially methylated genes

Gene	Correlation with *Fusobacterium*	LogFC	Adjusted *p* value
EGFR	0.299	0.581	0.035
PLCH1	0.297	0.646	0.035
COBL	0.296	0.491	0.038
mirLET7D	0.286	0.622	0.046
SFRP5	0.281	1.463	0.031

S5D. Pyrosequencing validation of selected candidate loci

Gene	Samples (T/NT)	Median methylation (%) T/NT	Mann-Whitney U	*p* value
PTPLAD2	36/3	53/37	36	0.300
SFRP5	45/3	46/42	59.5	0.701
CILP2	54/10	28/26	199	0.149
PLCH1	46/10	75/56	57	<0.001
mirLET7D	44/3	83/78	26.5	0.001
EGFR	42/10	43/40	130	0.040
COBL	54/10	77/70	114.5	0.002

S5E. Correlation and regression between relative *F. nucleatum* quantification and CpG island methylation percentage

Gene	Pearson r	Regression F	p value
EGFR	0.0001	0.015	0.904
PLCH1	0.104	0.593	0.444
COBL	-0.137	1.184	0.281
mirLET7D	0.136	0.845	0.363
SFRP5	-0.129	0.775	0.383
CILP2	0.022	0.031	0.861
PTPLAD2	0.108	0.435	0.514
ZNF788	0.278	5.179	0.026

S5F. Predictors retained in the linear regression model for ZNF788 methylation percentage

Predictor	Unstandardised coefficient	*p* value
Constant	31.73	
CIMP status	14.22	0.016
Relative *F. nucleatum* quantification	4.22	0.048
MSI status	-12.19	0.051

S5G. Variables excluded from the ZNF788 methylation model

Excluded variable	*p* value
Age	0.86
CRC stage	0.67
Tumour location	0.57
Sex	0.40

For S5B-S5C, LogFC denotes log2 fold-change between tumour and adjacent non-tumour mucosa; p values are adjusted for differential methylation. F. nucleatum qPCR correlations use normalised relative quantification.

Supplementary Table S6. Associations between *F. nucleatum* relative quantification and clinicopathological or molecular variables

S6A. Sex and tumour location

Comparison/variable	Mean difference	95% CI	t statistic	*p* value
Sex	-0.017	[-0.50; 0.46]	-0.07	0.94
Tumour location	0.15	[-0.38; 0.69]	-0.15	0.57

S6B. CRC subtype

Comparison	Mean difference	95% CI	t statistic	*p* value
Conventional vs Serrated	-0.68	[-1.21; -0.16]	-2.63	0.011
Conventional vs MSI	-1.01	[-1.62; -0.39]	-3.31	0.002
Serrated vs MSI	-0.32	[-1.13; 0.48]	-0.80	0.426

S6C. Local invasion category (T stage), Mann-Whitney U tests

Comparison	Mann-Whitney U	*p* value
Tis/T1/T2 vs T3	116	0.000021
Tis/T1/T2 vs T4	66	0.035
T3 vs T4	206	0.043

S6D. Overall CRC stage

Comparison	Mean difference	95% CI	t statistic	*p* value
0-I vs II	-1.75	[-2.43; -1.07]	-5.24	0.00001
0-I vs III	-1.47	[-2.11; -0.82]	-4.59	0.00004
0-I vs IV	-0.92	[-1.75; -0.10]	-2.36	0.03
II vs III	0.28	[-0.23; 0.79]	1.10	0.28
II vs IV	0.82	[0.06; 1.60]	2.19	0.04
III vs IV	0.545	[-0.19; 1.28]	1.50	0.14

S6E. MSI categories

Comparison	Mean difference	95% CI	t statistic	*p* value
MSS vs MSI-L	-0.34	[-0.93; 0.25]	-1.13	0.26
MSS vs MSI-H	-0.74	[-1.40; -0.08]	-2.24	0.03
MSI-L vs MSI-H	-0.40	[-1.33; 0.51]	-0.91	0.37

S6F. *KRAS* and *BRAF* status

Variable	Mean difference	95% CI	t statistic	*p* value
KRAS status	0.54	[0.02; 1.06]	2.07	0.042
BRAF status	-1.09	[-1.73; -0.46]	-3.43	0.001

S6G. CIMP categories

Comparison	Mean difference	95% CI	t statistic	*p* value
CIMP-negative vs CIMP-low	0.13	[-0.52; 0.79]	0.41	0.67
CIMP-negative vs CIMP-high	-0.62	[-1.25; 0.006]	-2.01	0.052
CIMP-low vs CIMP-high	-0.75	[-1.42; -0.08]	-2.28	0.027

S6H. *PIK3CA* status

Variable	Mean difference	95% CI	t statistic	*p* value
PIK3CA status	0.54	[-0.28; 1.37]	1.32	0.19

F. nucleatum was analysed as log-transformed normalised relative quantification.

Supplementary Table S7. Diagnostic performance and logistic regression models using *F. nucleatum* quantification

S7A. ROC performance for tumour versus adjacent non-tumour mucosa

AUC	95% CI lower	95% CI upper	Youden-optimised cutoff	Sensitivity	Specificity	Accuracy
0.678	0.594	0.763	ΔCt ≤ 10.2 cycles	47%	91%	69%

S7B. Logistic regression model for CRC detection, adjusted for age and sex

Predictor	Odds ratio	95% CI	*p* value
Relative *F. nucleatum* quantification	2.23	1.51-3.30	0.0001
Age	0.99	0.95-1.02	0.318
Sex	1.08	0.55-2.13	0.826

S7C. Logistic regression model for serrated versus conventional histology, adjusted for age and sex

Predictor	Odds ratio	95% CI	*p* value
Relative *F. nucleatum* quantification	2.00	1.22-3.28	0.006
Age	0.98	0.94-1.03	0.48
Sex	0.89	0.33-2.36	0.81

S7D. Best-fitting multivariable model for serrated versus conventional histology

Predictor	Odds ratio	95% CI	*p* value
Relative *F. nucleatum* quantification	5.91	1.94-18.03	0.002
Tumour location	0.19	0.04-0.99	0.049
KRAS status	7.10	1.06-47.54	0.043
PIK3CA status	0.01	<0.001-0.35	0.013

S7E. Variables excluded from the best-fitting multivariable model

Excluded variable	p value
BRAF	0.26
Sex	0.51
Age	0.63
CRC stage	0.66
CIMP status	0.85
MSI status	0.99

ROC cutoff is reported as the Youden-optimised difference in Ct values between F. nucleatum and total bacterial load. Logistic models use qPCR-based relative F. nucleatum quantification.

Supplementary Table S8. Primer sequences and assay designs for qPCR and pyrosequencing

Assay type	Target/locus	Forward primer (5'-3')	Reverse primer (5'-3')	Sequencing primer / probe
qPCR	Total bacteria (16S rRNA)	CGTGCCAGCCGCGG	TGGACTACCAGGGTATCTAATCCTG	N/A
qPCR	F. nucleatum	CAACCATTACTTTAACTCTACCATGTTCA	GTTGACTTTACAGAAGGAGATTATGTAAAAATC	N/A
qPCR	*ACTB* (Human beta-actin)	CCTCCATCGTCCACCGCAAATG	TGCTGTCACCTTCACCGTTCCA	N/A
Pyrosequencing	*PTPLAD2*	ATATAAAGATTAGGAAGTTGTAATTAGAGT	[Btn]AAATTCTCCAAATTTCTACAACACTACT	AGTTGTAATTAGAGTTTGGT
Pyrosequencing	*SFRP5*	TGTTTATTTTTTAGGTTTTGTGTTAGTAAT	[Btn]ACCCTACCAACTATACCTTACTTAC	TTAGGTTTTGTGTTAGTAATAA
Pyrosequencing	*CILP2*	TGGGATGTAGTTTGGAGGATTG	[Btn]CCCCTCTAAATCCCCCCCCTTAATAC	TTTGGAGGATTGAGTG
Pyrosequencing	*PLCH1*	GTGTTTGTTTTAGATGGAGTGATGT	[Btn]CCTCCTCACCTTCAAAAATCATT	ATGTTTATTTTTTGTATTTAGTTTG
Pyrosequencing	*mirLET7D*	TGTATTTTAGTTTGGGTGATAGAG	[Btn]TACTTAAAATCCAACCTCAAATCACAA	AAAATTGATAATTAAATGTTAAAAG
Pyrosequencing	*EGFR*	AGGGTGTTTGATAAGATTTGAAG	[Btn]CTTTATTCCAATCCCCACCTCCCCTAC	GATTTTAGGATTTTAGAGTATTATT
Pyrosequencing	*COBL*	GGGATTGGTTTTAGGATTTTTTATAGATA	[Btn]ACCTCAAATAATCTACCCACCTTAA	TGGTTTTAGGATTTTTTATAGATAT
Pyrosequencing	*ZNF788*	TTTTAAAGAGGAAGTGAATAGAATTAGTT	[Btn]CTCCTTCCCCCAAAAAAATTAACA	GTTGTTATTTAGGGTTTTGAA

[Btn]: 5’ Biotin-labeled

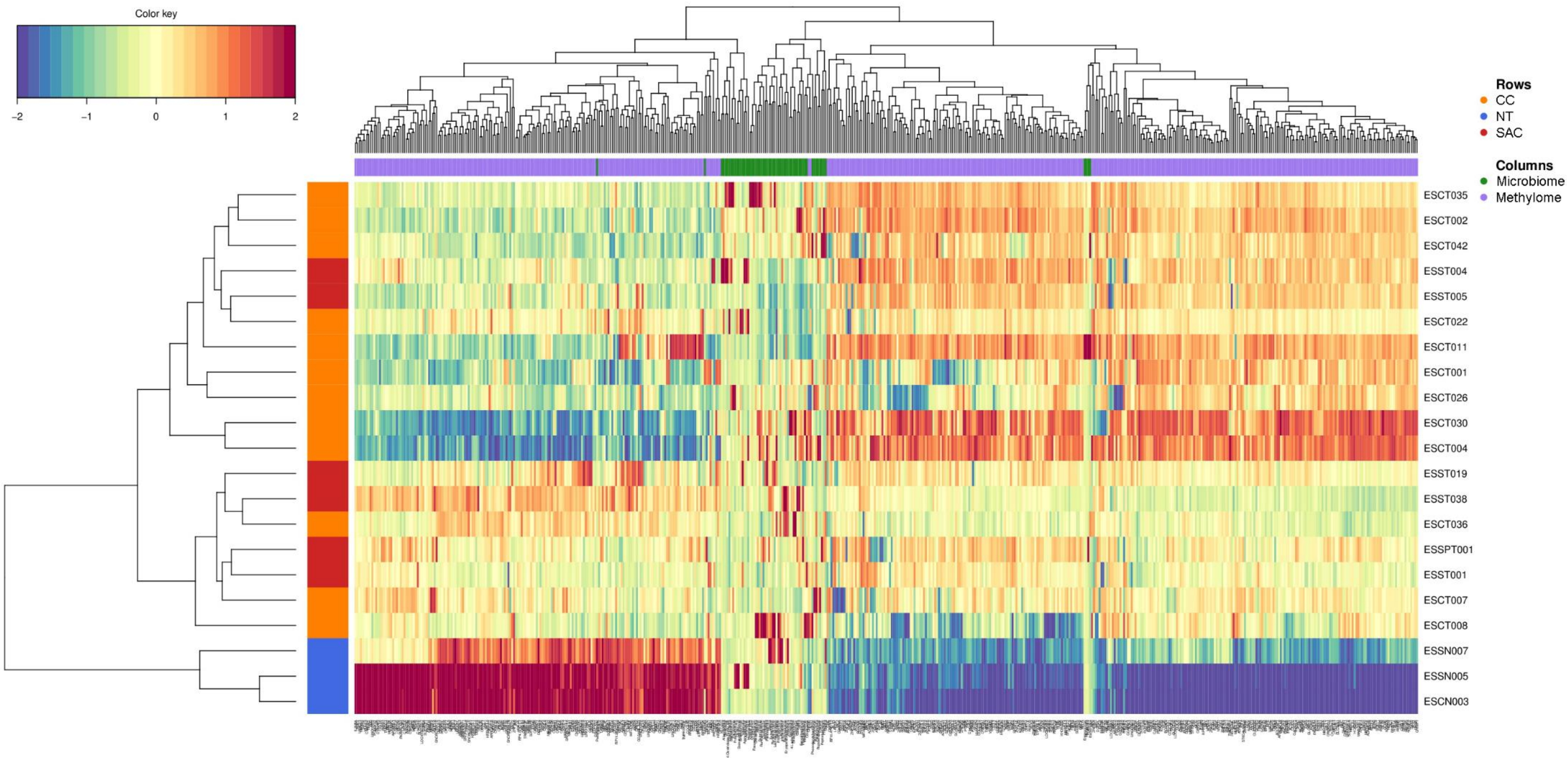

Figure S1. Heatmap of selected microbiome–methylome associations from multiblock sPLS-DA. Clustered image map showing selected microbial and methylation features retained by the DIABLO/sPLS-DA model. Rows and columns represent features selected from the microbiome and methylome blocks, respectively, and colour intensity indicates the strength and direction of the association between selected features.

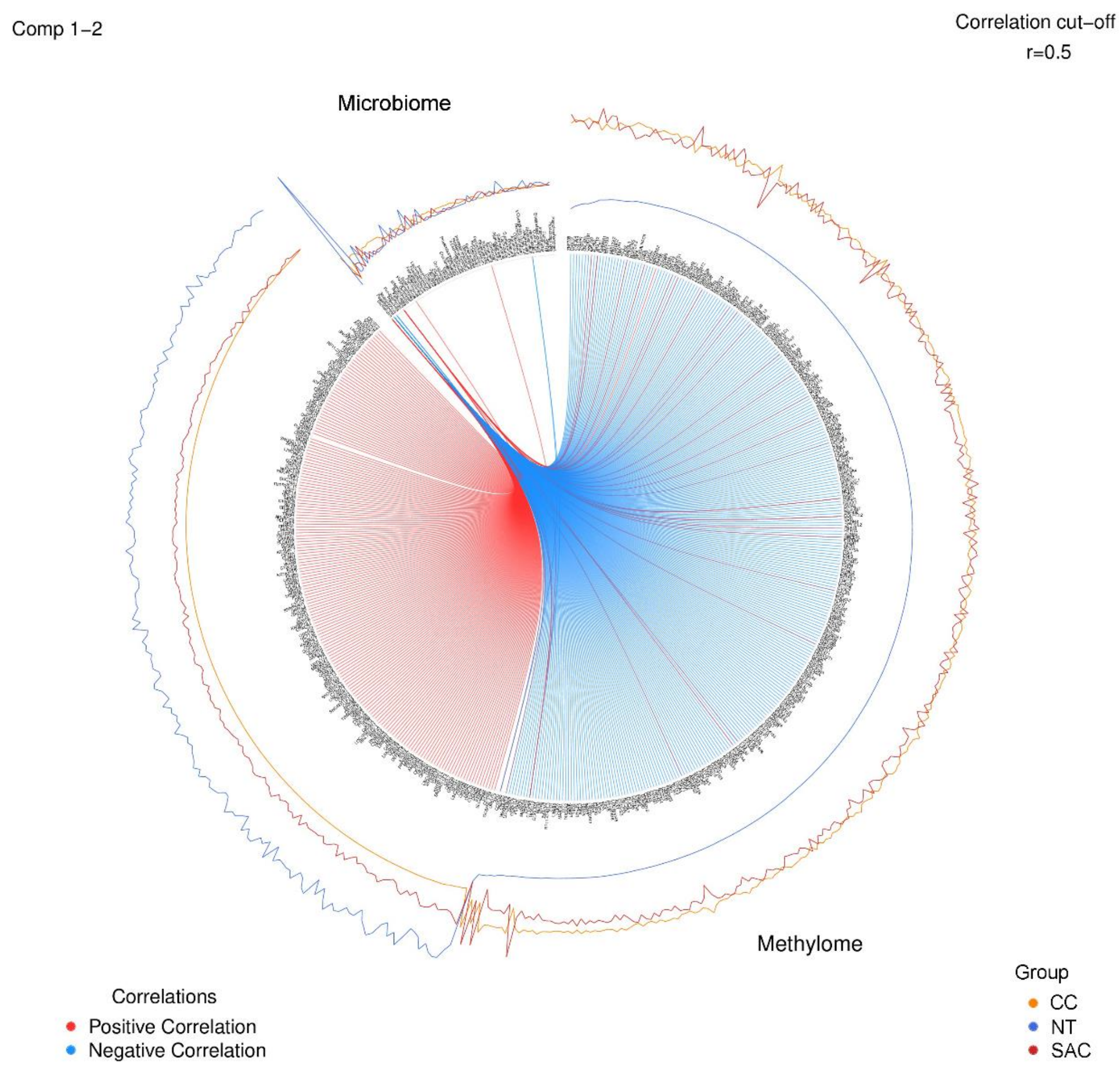

Figure S2. Circos plot of selected cross-omic associations from multiblock sPLS-DA. Circos representation showing selected associations between microbial taxa and methylation features retained by the DIABLO/sPLS-DA model. Links connect features across omics blocks, with line intensity reflecting the strength of the association.
